# The Characteristics of Time-Dependent Changes of Coefficient of Permeability for Superabsorbent Polymer-Soil Mixtures

**DOI:** 10.3390/ma15134465

**Published:** 2022-06-24

**Authors:** Jakub Misiewicz, Sujit Sankar Datta, Krzysztof Lejcuś, Daria Marczak

**Affiliations:** 1 Institute of Environmental Engineering, Wrocław University of Environmental and Life Sciences, 50-363 Wrocław, Poland; jakub.misiewicz@upwr.edu.pl (J.M.); daria.marczak@upwr.edu.pl (D.M.); 2 Department of Chemical and Biological Engineering, Princeton University, Princeton, NJ 08544, USA; ssdatta@princeton.edu

**Keywords:** superabsorbent polymers, coefficient of permeability, infiltration, flow rate, SAP-soil mixture

## Abstract

Water uptake dynamics of superabsorbent polymers (SAP) in soil is of key importance for the optimum application of these materials in environmental engineering and agriculture, so goal of this paper is to determine time dependent values of coefficient of permeability for various SAP-soil mixtures. Retaining water in soil is a key requirement in critical zones to support plant growth. There is an urgent need for technologies that can increase soil water retention, given the increasing prevalence of droughts and scarcity of clean water as the climate changes, combined with the rising demand for food by a growing world population. SAPs are materials that can absorb significant amounts of water, and thus have tremendous potential to help increase water retention in soil. However, while some studies have characterized the equilibrium swelling behavior of SAPs in soil, how their addition influences the time-dependent flow of water through soil remains poorly understood. Here, we address this gap in knowledge by directly measuring the coefficient of permeability of SAP-soil mixtures, testing different soil grain sizes, SAP grain sizes, and different SAP-soil ratios. We find that SAP addition can dramatically hinder the flow rate of water through soil—reducing the permeability by several orders of magnitude, and in some cases causing complete blockage of water infiltration, at mass fractions as small as 1%. In this scenario coefficient of permeability of 1.23 × 10^−4^ m/s dropped by a factor of ~10 after 14 min, a factor of ~100 after 36 min, and by nearly a factor of ~1000 after 63 min, eventually causing complete blockage of infiltration after 67 min. Authors concluded that in this particular situation the size and quantity of SAP particles was enough to nearly completely fill the available pore space resulting in rendering the soil column almost completely impermeable. Moreover, we demonstrate that these effects are well-described by a simple hydraulic model of the mutual interactions between SAP and soil grains, providing more generally-applicable and quantitative principles to model SAP-soil permeability in applications. Ultimately, this work could help evaluate the optimal proportions and grain sizes of SAPs to use for a given soil to simultaneously achieve a desirable permeability along with increased water holding capacity in the plant root zone.

## 1. Introduction

Superabsorbent polymers (SAPs) are promising for environmental uses as reservoirs of water in dry soil, potentially alleviating the burden of irrigation [1]. However, confinement in soil can markedly reduce the ability of superabsorbents to absorb water and swell, and influence water flow through soil profile [2]. Thus, real-world uses of these materials proceed by trial and error, yielding highly variable results that limit the widespread adoption of superabsorbents in agriculture and environmental engineering [3]. Scientific goal of this paper is to describe time dependent characteristics of water flow through soil profile influenced by various quantities of SAP addition, taking into account various grain size distributions of used materials.

Water plays a key role everywhere where plant growth is concerned. Optimum management of this resource is particularly important from the point of view of agricultural production or biotechnological solutions used in environmental engineering [4,5]. The physicochemical properties of the upper soil layer will determine the mutual interactions between water infiltration, runoff, and evaporation under given climatic conditions, and therefore influence its usability for supporting plant growth [6,7]. Hence, in recent decades, grains of super-absorbent polymers (SAP) have attracted increasing interest as soil additives to rationally tune these properties and to stabilize the soil [8,9,10]. In particular, the high number of hydrophilic groups in the polymer chains contributes to their ability to absorb water in amounts multiple times higher than their own mass [11,12]. Additionally, their three-dimensional, crosslinked structure ensures the capacity to retain water from rainfall or irrigation systems to be gradually absorbed by plant roots supporting their growth [13,14]. Apart from improving the water conditions in soil, SAPs are also applied as additives for controlled release of fertilizers, increasing their absorption by plants and slowing down infiltration into the deeper soil layers, thereby reducing groundwater pollution [15,16]. The application of SAPs as soil additives is therefore considered to be an effective strategy improving the conditions of plant vegetation in soil, such as water retention, nutrients, infiltration, aeration, or improved structure of the substrate, which, in turn, has a positive influence on crops [17,18]. It is reasonable to point out that the effects of SAP amendments on plant vegetation may be site specific and vary depending on what measure of the plant community is being assessed [19]. Better understanding water transport in SAP-soil mixtures is therefore practically important in agriculture and to our environment.

Unfortunately, previous studies of the interactions between SAPs and soil have yielded conflicting results [20,21]. Some of the studies point to a significant increase in crop growth with reduced water consumption for irrigation (by 40%) [22,23] and overall increase of available water content for plants up to 5 times after the application of SAP [24]; conversely, some other publications point to less beneficial results, where the addition of SAP led to only an initial improvement of plant vegetation conditions [19] or instead had a negative influence on strength parameters of soil. When the content of SAP reaches a certain limit, the particles of SAP existing in the voids of soil could absorb excessive amount of water and damage the skeleton of the soil due to volumetric expansions, resulting in deterioration in the interlocking and bonding between soil particles [25]. Addition of SAP can also alter soil structure and pore geometry by clogging the larger pore in coarse textured soil [26].

However, systematic laboratory experiments are beginning to unravel the origins of these conflicting results. One factor that is now appreciated to play a key role is the pressure experienced by the SAP inside soil: in real-world applications, SAP swelling upon contact with water is highly dependent on the local pressure [3,27,28]. Indeed, SAP absorption of water has been shown to be dramatically reduced upon simply loading a SAP-soil mixture with a top layer of soil, due to the increased pressure inside [29]. This effect is often missed in laboratory tests that either neglect the influence of load caused by the top layer of soil [30,31], or analyze the absorbency under load of SAP grains only in the absence of soil [32,33]. In reality, regardless of the manner of introducing the SAP into soil, its swelling will be limited by the load of the topsoil layer and by the available pore space [34]. The suitability of the SAP to the given soil should be based on such geometric parameters of soil structure as the size, shape, and placement of individual grains, as well as on such physical properties as soil bulk density or porosity [35]. During swelling, the SAP-soil mixture exerts pressure on the topsoil layer. The value of this pressure is closely linked to the dose of SAP, the size of its grains, and the available pore space.

While the equilibrium amount of SAP absorption and its dependence on lo-cal pressure is now better understood, less well-understood is the time required to obtain the maximum water absorption and pressure values and thus, to achieve a state of mechanical equilibrium [36,37]. Soil structure is determined by the distribution of various soil grain size fractions and it represents the basic characteristics of soil, which directly influences the physical, chemical, and biological processes that take place in soil [35]. Research experiments also demonstrate that the soil grain size distribution may influence the infiltration of water into soil, water retention, as well as the availability of water and nutrients for plants. Numerous model studies also point to the influence of the soil grain size distribution on the dynamics of degradation of the organic matter that is present in soil [38,39]. The results reveal that smaller grain sizes are characterized by stronger spatial capacity to fill the pores of soil, which corresponds to higher values of fractal dimensions based on pore geometry [40]. Various concentrations of SAP additives in soil have a significant influence on the structural distribution of its grains. Along with the increase in the amount of SAP, the fractal dimension of the pore space becomes smaller, and the volume of pores decreases significantly. The concentration of SAP in soil is negatively correlated with the pore space fractal dimension of the clay-particle fraction, silt volume fraction, and positively correlated with the sand volume fraction [41]. Given these effects, better understanding the water uptake dynamics of SAPs in soil is of key importance for the optimum application of SAP-soil mixtures in environmental engineering and agriculture, for which the dynamics and swelling of these materials and their interactions with soil are critically important.

In addition to the water uptake and swelling dynamics of SAPs in soil, another critically important property is their influence on the permeability of soil to water. Indeed, water infiltration in soil is a key element of the hydrological cycle that mainly controls the absorption of water by the soil profile and its discharge [42]. High values of the coefficient of permeability and water penetration outside the plant root zone are characteristic for coarse-textured soils (sand, sandy loam etc.) [43]. On the other hand, clayey soils with a low coefficient of permeability are prone to the creation of impermeable lenses that result in flooding the land and disturbing the optimal water-to-air ratio, which may cause waterlogging [44,45]. These dynamics are likely to be strongly dependent on the grain size distribution of SAP as well as the size of available soil pores, given that SAPs achieve the state of swelling balance in a porous medium and under load in a different way than in free water absorption conditions—but how remains unclear.

Given these gaps in knowledge, here, we built on our previous studies [3,27,28] to characterize how the addition of SAP to soil changes its coefficient of permeability over time, which is a novel approach, and how these dynamics depend on SAP grain size and soil grain size. We found that even small amounts of SAP can dramatically decrease the soil permeability, in some cases even causing complete blockage of water infiltration that can have important consequences in real-world settings. Moreover, we showed that the permeability measurements can be described using a simple physical model of the soil permeability. Altogether, this work provides useful new data and modelling that will be of importance in the field of materials used in water resources management.

## 2. Materials and Methods

**Figure 1:**
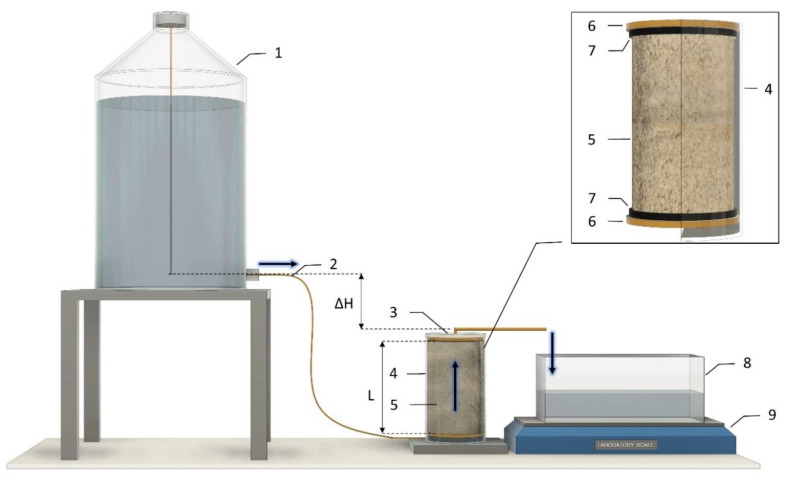
Schematic drawing of the test equipment, where: 1. Mariotte’s bottle; 2. Elastic tube; 3. Sealed top cover with an outflow tube; 4. Stainless steel cylinder with a watertight bottom; 5. SAP-soil mixture; 6. Porous pad; 7. Filtration mesh disc; 8. Plastic container; 9. Weighing scale machine.

The analysis of changes in the coefficient of permeability was conducted at the Soil Testing Laboratory of the Institute of Environmental Engineering of the Wroclaw University of Environmental and Life Sciences, with use of the specially-designed laboratory setup shown in Figure 1.

The tests were conducted using three classes of materials:

- Two types of soils collected in the field—Coarse sand and Loamy sand;
- Two types of commercially available superabsorbents- cross-linked copolymer of acrylamide and potassium acrylate: Aquasorb 3005 KS and Aquasorb 3005 KM (hereafter referred to as KS and KM, respectively), obtained from SNF FLOERGER, Andrézieux, France;
- Substance that will infiltrate through the samples—deionized (DI) water.

The grain size distribution of the SAPs and soils used in this study was determined by the sieving method. For each of the analyzed materials, 500 g of air-dry sample was sieved through a set of 6 sieves with mesh widths of 0.10, 0.25, 0.50, 1.00, 2.00 and 5.00 mm. For loamy sand, which consisted of smaller grains, hydrometer analysis was conducted. The analysis of the KS superabsorbent was performed in the Mastersizer 2000 laser granulometer (Malvern Instruments Ltd.; Malvern Panalytical Ltd., Malvern, UK) with the SCIROCCO 2000 module. Measurements were taken on dry aerosol, at the pressure of 2 bars with the measured grain diameter ranging from 0.02 to 2000 microns. Each test was conducted 3 times, and the results were then averaged. As a result, grain size distribution histograms with a range of grains were created. Basic properties of the soil and SAP used for study are presented in Table 1. The tested soils were classified according to the USDA (United States Department of Agriculture and the National Cooperative Soil Survey) classification.

The laboratory setup preparation consisted of the following steps:

- SAP-soil mixture in the air-dry state was prepared at a predefined proportion (0.3%, 0.5%, or 1.0% of SAP addition by mass);
- A mixture was placed in a stainless-steel cylinder (volume *V* = 760.0 cm^3^, height *L* = 8.0 cm, diameter *D* = 11.0 cm) having a porous base with a rapid filtering grade mesh;
- A Cylinder containing the SAP-soil mixture was placed on a vibration plate in order to achieve the desired sample dry bulk density (*ρ*_d_ = 1.65 g/cm^3^);
- A filtration mesh and porous circular pad was placed on top of the SAP-soil mixture.
- The laboratory equipment (Figure 1) was arranged in the following order: Mariotte’s bottle filled with water placed at height *H*_1,_ connected with the cylinder containing the SAP-soil mixture of a defined height *L*, covered with a lid with a water outlet tube (*D* = 0.4 cm, *L* = 15 cm) at the defined height *H*_2_, leading to the container placed on the weighing scale machine.

### 2.1. Coefficient of Permeability Measurement Procedure

The test begins with opening the valve on the Mariotte’s bottle, which leads to water flow from the whole system through the SAP-soil sample with constant head and hydraulic gradient (Equation (1)). Taring the scale with the container placed on it begins the recording of the reading at 1 min intervals for the whole duration of water flow through the analyzed material which is 120 min. The test is considered to be finished after a constant increase in water weight in time has been achieved. For each increase in weight during the interval, the transient value of the coefficient of permeability *K*_t_ (Equation (2)) at the given temperature *T* was calculated. Then, taking into account the value of dynamic viscosity of water at 10 °C flowing through the sample, as is conventionally carried out, the value of the coefficient of permeability (*K*_10_) was calculated using Equation (3).

*i* = Δ*H*/*L* [-] (1)

*K*_t_ = *Q*/(*A* · *i*) [m/s] (2)

*K*_10_ = *K*_t_/(0.7 + 0.03 · *T*) [m/s] (3)

where *Q* is the volumetric flow rate of water [cm^3^ ⋅min^−1^], *A* is the cross-sectional area of the SAP-soil sample [cm^2^], *i* is the hydraulic gradient [-], and *T* is the water temperature [°C], *L* is the height of the sample [cm], and Δ*H* is the height difference between the outflows from the Mariotte’s bottle and the stainless-steel cylinder [cm]. 

Using this protocol, in each experiment testing different SAP and soil grain sizes, we characterized changes in the value of the coefficient of permeability at 10 °C over time. The experiments on each SAP-soil mixture were repeated three times, and the results were averaged.

### 2.2. Physical Characteristics of SAP and Soil Grains

**Figure 2:**
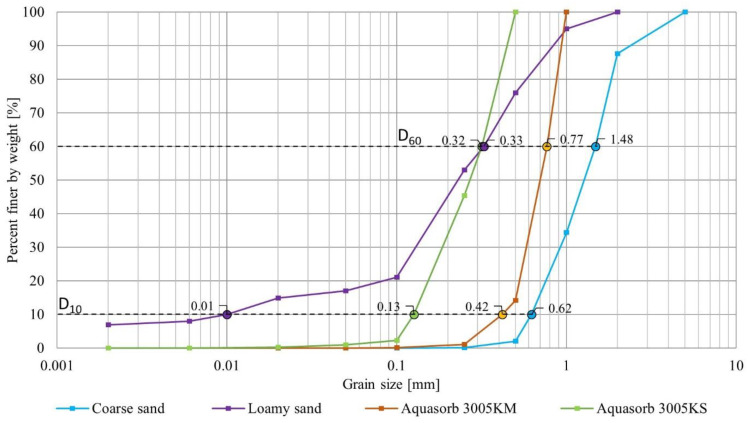
Grain size distributions of the tested SAP and soil.

The basic physical parameters of materials used in this study were analyzed to determine the structural characteristics of the SAP-soil mixtures. The factors are particularly important in practical applications involving water infiltration: the size ratio between dry SAP and soil grains, the porosity quantifying the available volume for SAP swelling (Equation (4)), and the sample bulk density. These parameters are presented in Table 1. Figure 2 presents the grain size distributions of dry SAPs and soils.

(4) $$n=1\;-\;\;\frac{\rho d}{\rho s}\;[-]$$

where, *n* is the soil porosity, ρ_d_ is soil bulk density, and ρ_s_ is soil specific weight.

For the Aquasorb 3005 KM, grains in the 0.5–1.0 mm diameter size range comprised the largest mass fraction of the sample (86%), followed by those in the 0.25–0.50 mm range (13%); the mean diameter was ~0.93 mm. Aquasorb 3005 KS was characterized mostly by grain sizes between 0.25–0.50 mm (55%) and 0.10–0.25 mm (43%), with a mean diameter of ~0.38 mm. Coarse sand was characterized with a less homogenous grain size distribution, predominantly with 1.00–2.00 mm diameter grains (53%), followed by 0.50–1.00 mm (33%) and 2.00–5.00 mm (12%); the mean diameter was 2.01 mm. Loamy sand was characterized by the broadest distribution of grain sizes, with grain diameters in the ranges 0.10–0.25 mm (32%), 0.25–0.50 mm (23%), 0.50–1.00 mm (19%), and 1.00–2.00 mm (5%); the mean diameter was 0.49 mm.

## 3. Results of Coefficient of Permeability Measurements

**Figure 3:**
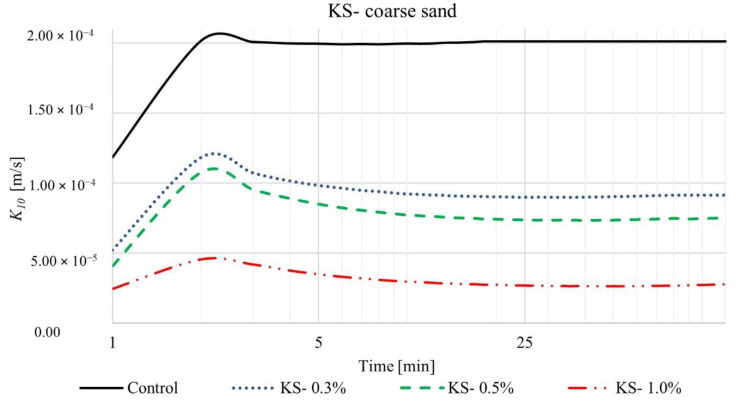
Time-dependent values of coefficient of permeability for SAP-KS and coarse sand mixtures, in proportions of 0.3, 0.5, and 1.0%.

**Figure 4:**
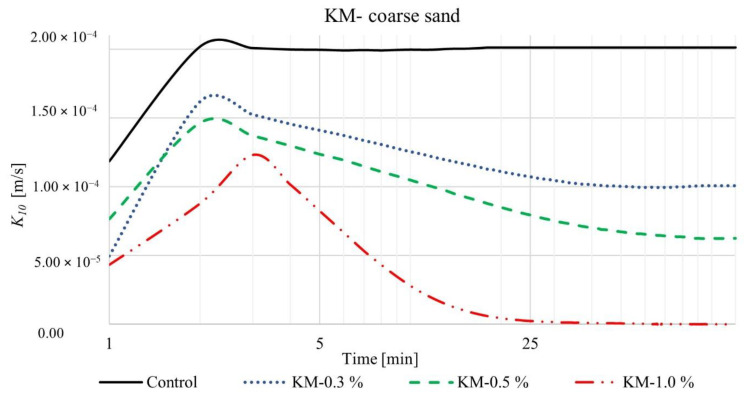
Time-dependent values of coefficient of permeability for SAP-KM and coarse sand mixtures, in proportions of 0.3, 0.5, and 1.0%.

**Figure 5:**
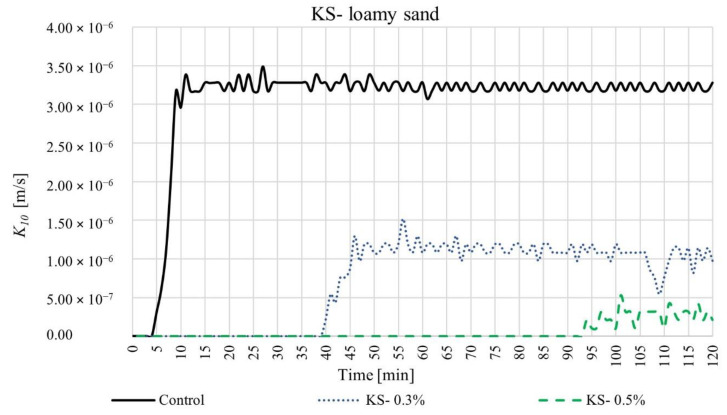
Time-dependent values of coefficient of permeability for SAP-KS and loamy mixtures, in proportions of 0.3, 0.5, and 1.0%.

**Figure 6:**
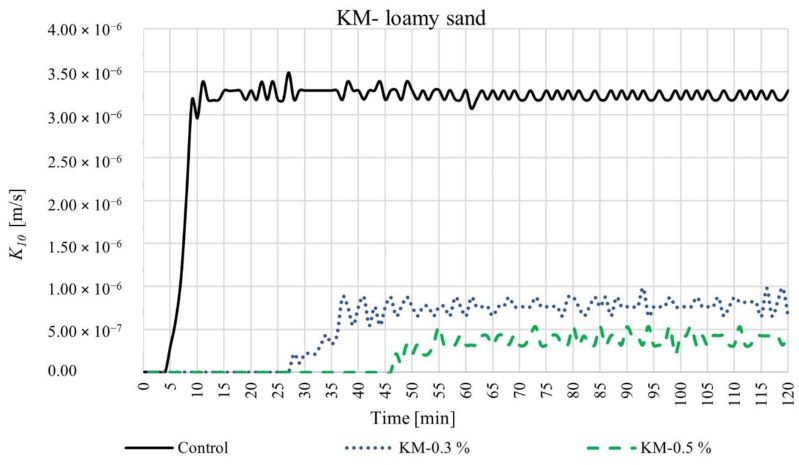
Time-dependent values of coefficient of permeability for SAP-KM and loamy mixtures, in proportions of 0.3, 0.5, and 1.0%.

We measured the coefficient of permeability over time for four different SAP-soil mixtures with increasing dry SAP-soil mean grain diameter ratios: KS-coarse sand (diameter ratio ~0.2), KM-coarse sand (diameter ratio ~0.5), KS-loamy sand (diameter ratio ~1), and KM-loamy sand (diameter ratio ~3). The results are shown in Figure 3, Figure 4, Figure 5 and Figure 6, respectively. For each such mixture, we tested three different SAP-soil proportions: 0.3, 0.5, and 1.0% by dry mass.

The results in Figure 3 and Figure 4 reveal that introduction of SAPs causes a dramatic decrease in the coefficient of permeability of soil. Indeed, small changes in the relative proportions of SAPs and soil have a noticeable influence on the permeability. Consider, for example, the coarse sand control group (without any SAP): the coefficient of permeability *K*_10_ stabilized almost immediately (2 min) after the beginning of the experiment and reached a steady-state value of 2.01 × 10^−4^ m/s (black lines in Figure 3 and Figure 4). Upon adding SAP-KS, we observed that the coefficient of permeability initially increased just as rapidly for ~2 min, reflecting water flow through the column, reaching maximal values of 1.18 × 10^−4^, 1.10 × 10^−4^, and 4.60 × 10^−5^ m/s for 0.3, 0.5, and 1.0% SAP-soil mixtures, respectively (shown by the peak in the blue, green, red lines in Figure 3). As the SAP grains absorbed water and swelled within the pore space, they reduced the permeability, causing it to reach a lower steady-state value after ~13 min: 9.14 × 10^−5^, 7.5 × 10^−5^, and 2.8 × 10^−5^ m/s for 0.3, 0.5, and 1.0% SAP-soil mixtures, respectively. We observed similar behavior for the SAP-KM, as shown in Figure 4. In particular, the coefficient of permeability reached maximal values after 2 min of 1.62 × 10^−4^, 1.46 × 10^−4^, and 1.23 × 10^−4^ m/s for 0.3, 0.5, and 1.0% SAP-soil fractions, respectively, corresponding to 27.16%, 24.66%, 62.60% faster water flow in comparison to SAP-KS samples. Again, as the SAP absorbed water and occupied a larger fraction of the pore space, they reduced the coefficient of permeability, causing it to reach a lower steady-state value after ~40–90 min: 1.01 × 10^−4^ and 6.26 × 10^−5^ m/s for 0.3 and 0.5% SAP-soil mixtures, respectively. For the largest SAP fraction of 1.0%, the maximal *K*_10_ of 1.23 × 10^−4^ m/s dropped by a factor of ~10 after 14 min, a factor of ~100 after 36 min, and by nearly a factor of ~1000 after 63 min, eventually causing complete blockage of infiltration after 67 min. Authors concluded that in this particular situation the size and quantity of SAP particles was enough to nearly completely fill the available pore space resulting in rendering the soil column almost completely impermeable.

The experiments with loamy sand, which had larger dry SAP-soil grain size ratios, exhibited even more dramatic changes in permeability, as shown in Figure 5 and Figure 6. The loamy sand control group (without any SAP) showed a 5 min delay between beginning the experiment and water beginning to flow out from the 8 cm-high sample (black line)—reflecting the lower permeability of the loamy sand as compared to the coarse sand. The coefficient of permeability then stabilized after ~15 min at a steady-state value *K*_10_ = 3.28 × 10^−6^ m/s. SAP addition again caused a marked decrease in permeability. Upon adding 0.3% SAP-KS or SAP-KM, we observed that the initial delay time was extended to 40 min or 28 min, respectively (blue lines in Figure 5 and Figure 6). The coefficient of permeability eventually reached steady-state values of *K*_10_ = 9.78 × 10^−7^ or 7.62 × 10^−7^ m/s for SAP-KS or SAP-KM after 120 or 38 min, respectively. These reductions in permeability were exacerbated for the case of 0.5% SAP addition: for SAP-KS or SAP-KM, we found initial delay times of 94 or 47 min, respectively (green lines in Figure 5 and Figure 6), with steady-state values of *K*_10_ = 3.18 × 10^−7^ or 4.23 × 10^−7^ m/s after 102 or 59 min, respectively. Indeed, in both SAP-KS and SAP-KM tests, SAP-soil fractions of 1.0% yielded no observable water outflow over the 120 min duration of the experiment: SAP addition rendered the soil column completely impermeable.

Our measurements have thus revealed the pivotal influence played by SAP swelling on the permeability of soil: adding ≤1% of SAP causes marked reductions in the coefficient of permeability, in some cases causing it to fall below the measurement resolution. What is the physical origin of these permeability reductions, and can they be modeled in a simple, generally-applicable way? To address this question, we consider a simple hydraulic model of permeability, following the pioneering work of Carman [46] and Verneuil and Durian [47]. In particular, we model the pore space through which water can flow as a bundle of parallel tubes with the same hydraulic radius *R*; the SAP-free soil hydraulic permeability $k_{\mathrm{control}}$ then scales as $\sim \varepsilon_{\mathrm{control}}R^{2}/\tau_{\mathrm{control}}$*,* where $\varepsilon_{\mathrm{control}}=0.38$ is the porosity and $\tau_{\mathrm{control}}$ is the pore space tortuosity. To describe how this permeability is reduced by SAP swelling in the pore space, we first note that without confinement in the soil matrix, the volume of each SAP grain increases by as much as ≈350 upon free water absorption [48]. Therefore, the volume ratio between fully-swollen SAP grains and the soil grains in our experiments ranged from ≈3 to ≈10^4^, suggesting that swollen SAP grains can clog open pores. Indeed, our direct visualization of this process [3] indicates that even though within a given pore, a swelling SAP grain eventually clogs the pore and its swelling is hindered within it, the SAP can continue to swell through the surrounding interstices into adjacent pores. We therefore assume as a first idealization step that swollen SAP grains within a soil column are spherical, uniform in size, and eventually adopt their fully-hydrated diameters within the pore space. A given SAP-soil mixture has $N_{\mathrm{SAP}}$ and $N_{\mathrm{soil}}$ grains of each, with individual grain dry mass densities $\rho_{\mathrm{SAP},\mathrm{dry}}$ and $\rho_{\mathrm{soil}}$ and dry volumes given by $\nu_{\mathrm{SAP},\mathrm{dry}}$ and $\nu_{\mathrm{soil}}$, respectively, and the individual grain volume of a swollen SAP is given by $\nu_{\mathrm{SAP}}\approx 350\;\nu_{\mathrm{SAP},\mathrm{dry}}$. The dry mass fraction (which in our experiments is 0.3, 0.5, and 1.0%) is then

(5) $$\xi \equiv \frac{N_{\mathrm{SAP}}\cdot \rho_{\mathrm{SAP},\mathrm{dry}}\cdot \nu_{\mathrm{SAP},\mathrm{dry}}}{N_{\mathrm{SAP}}\cdot \rho_{\mathrm{SAP},\mathrm{dry}}\cdot \nu_{\mathrm{SAP},\mathrm{dry}}+N_{\mathrm{soil}}\cdot \rho_{\mathrm{soil}}\cdot \nu_{\mathrm{soil}}}$$

Based on the bulk density and our previous measurements [27,28], we take $\rho_{\mathrm{SAP},\mathrm{dry}}$ ≈ 1.1 g/cm^3^ and $\rho_{\mathrm{soil}}$ ≈ 2.65 g/cm^3^; thus,

(6) $$\frac{N_{\mathrm{SAP}}}{N_{\mathrm{soil}}}\approx \frac{840\xi }{1-\xi }\cdot \frac{\nu_{\mathrm{soil}}}{\nu_{\mathrm{SAP}}}$$

Furthermore, since the internal permeability of the swollen SAP is much smaller than the permeability of the soil matrix [49] we treat the SAP as being completely impermeable to water flow. Then, computing the probability that a given point within the SAP-soil mixture is encompassed by a randomly-placed swollen SAP grain [50] yields the new porosity of the SAP-soil mixture,

(7) $$\varepsilon =\varepsilon_{\mathrm{control}}e^{-\varphi }$$

where $\varphi =V_{\mathrm{SAP}}/V$ is the total volume fraction occupied by swollen SAP with $V_{\mathrm{SAP}}=N_{\mathrm{SAP}}\nu_{\mathrm{SAP}}$ and $V\approx N_{\mathrm{soil}}\nu_{\mathrm{soil}}/\left(1-\varepsilon_{\mathrm{control}}\right)$ representing the total swollen SAP volume (neglecting the soil grains) and the total system volume, respectively. 

Finally, assuming for simplicity that the hydraulic radius *R* is set by the soil grain size and is therefore unchanged upon SAP addition, and that the pore space tortuosity is also unchanged upon SAP addition, then yields our final approximate prediction for the SAP-soil mixture permeability *k*:

(8) $$\frac{k}{k_{0}}=\frac{K_{10}}{K_{10,\mathrm{control}}}\approx \exp\;\left[\frac{-840\xi \left(1-\varepsilon_{0}\right)}{1-\xi }\right]$$

where $K_{10,\mathrm{control}}$ represents the coefficient of permeability for the SAP-free control case of just a soil packing. 

**Figure 7:**
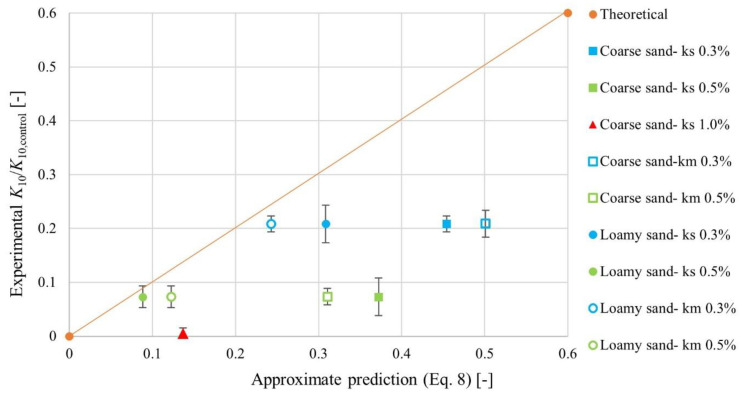
Comparison between experimental measurements and the theoretical prediction of Equation (8). The vertical axis shows the long-time coefficient of permeability *K*_10_ obtained by averaging the last 20 min of each test and normalized by the corresponding value for the SAP-free control case, *K*_10,control_. The horizontal axis shows the corresponding theoretical prediction. The two agree to within a factor of ~3.

As shown in Figure 7, despite the many simplifying assumptions inherent in this calculation, our prediction quantified by Equation (8) works remarkably well in describing the variation of the coefficient of permeability with increasing SAP addition, agreeing with the experimentally-measured steady-state values to within a factor of ~3. Similar agreement was observed in prior experiments using model SAP-glass bead mixtures [47,51]; our measurements provide a direct extension to more realistic SAP-soil mixtures, suggesting that this simple model can be reasonably applied to field settings, as well. As expected, we find particularly good agreement at the lower SAP fractions. Intriguingly, we find better agreement between the measurements and the theoretical prediction for loamy sand with smaller pore sizes.

## 4. Discussion

In this study, the influence of SAP addition on soil permeability and its dynamics influenced by SAP swelling was validated. We tested SAP-soil mixtures in conditions of constant sample volume, so that swelling of SAP grains was possible only in the porous matrix of soil. This refers to the situation where SAP-soil mixture is fully constrained by the topsoil layer and the upward expansion is not allowed. In this case, we found that SAP addition to soil can drastically decrease the rate of infiltration, potentially keeping water in the topsoil layer for longer and slowing down water runoff [25]. Too much SAP (e.g., 1% KM) can even cause complete blockage of soil pores and thereby render the mixture effectively impermeable. We observed that the maximal coefficient of permeability value *K*_10_ of 1.23 × 10^−4^ m/s for coarse sand mixed with SAP dropped by a factor of ~1000 after 63 min of experiment which clearly showed unintuitive limitations. These results thus help to rationalize previous measurements in which adding sodium polyacrylate to soil resulted in completely hindered water infiltration into the deeper soil layers considerably restraining the migration of moisture in the soil [52], and other measurements in which SAP-amended soil was shown to have a drastically-slower vertical infiltration rate of water [53]. Our findings also help to rationalize observations of an increase in the soil moisture up to 400% at field capacity upon 2% SAP addition [54]. On top of that, our findings shows direct correlation between grain size distribution of used materials and pace of water flow through the sample. All tested proportions of SAP KS and coarse sand mixtures are characterized by fast stabilization of infiltration process, by the mean grain size diameter ratio 0.38/2.01 mm. Progressive fall of obtained permeability results is observed for SAP KM mixed with coarse sand (mean grain size parameters ratio = 0.93/2.01 mm), leading to complete blockage of soil pores. This resonates directly with our previous findings that shows connection between grain size of used SAPs and values of swelling pressure [28]. Our model provides a simple, generally-applicable way of quantifying these effects. Despite its simplicity, it captures our measurements reasonably well; future extensions could consider variations in the porosity, sphericity, angularity, and roughness of individual SAP and soil granules, and also dynamic changes is sample height caused by absorbency under load [27], given that these parameters alone can influence the coefficient of permeability values substantially [55]

## 5. Conclusions

Water absorbing soil amendments researched in this paper have a great potential to be used in various practical applications as water reservoir for plants, especially in soils with low water holding capacity. This study shows that SAPs can drastically decrease the rate of water infiltration through soil profile. Control samples for both tested soils, coarse sand and loamy sand are characterized by coefficients of permeability of *K*_10_ = 2.01 × 10^−4^ m/s and *K*_10_ = 3.28 × 10^−6^ m/s, respectively, and they reach those values as fast as 2 min and 15 min from the beginning of experiment. Addition of swelling particles radically delays reaching steady-state values for all tested scenarios. Experimental data confirm that the size of SAP granules is important parameter that indicates its usability with various soils. Also, the higher amount of SAP is added to the mixture, the slower pace of water flow is observed. In case of fine granule soil- loamy sand, the SAP addition lowered overall value of coefficient of permeability to *K*_10_ = 9.78 × 10^−7^ or 7.62 × 10^−7^ m/s for SAP-KS or SAP-KM but reached steady-state values after 120 or 38 min, respectively. In the case of loamy sand mixed with 1% addition of SAP, in both cases (KM and KS) water flow was hindered completely for 120 min duration of experiments. SAP addition to coarse grained soil- coarse sand can be an effective method to slow down the water infiltration and hold moisture in plant root zone. But the experiments showed that there are some unintuitive limitations (case of 1% addition of KM to coarse sand) which can lead to opposite effect to the one intended. In conditions where soil sample with SAP addition is fully constrained and upward movement is not possible, we observed a near complete blockage of the soil pores. So, in practical applications topsoil layer thickness can be a limiting factor for optimum use of these materials. This paper shows an innovative way to test various swelling water absorbing materials blended in soil matrix, by precise, time dependent measurements. Moreover, we showed that the permeability measurements can be described using a simple physical model of the soil permeability. Altogether, this work provides useful new data and modelling that will be of importance in the field of materials used in water resources management.

## Figures and Tables

**Table 1:** Basic properties of the soils and SAPs used for study.

Physical Properties		Soil/SAP			
		Coarse Sand	Loamy Sand	KM	KS
Specific Weight [g/cm^3^]		2.65	2.65	1.10	1.10
Bulk Density [g/cm^3^]		1.65	1.65	NA	NA
Porosity [-]		0.38	0.38	NA	NA
Grain Size Distribution [%]	Gravel (>2.00 mm)	12.34	0	0	0
	Sand (0.05 mm–2.00 mm)	87.66	85.01	100	98.96
	Silt (0.002 mm–0.05 mm)	0	14.99	0	1.04

NA, not applicable/negligible.

## Data Availability

Not applicable.
